# Editorial

**Published:** 1862-06

**Authors:** 


### Editorial.

Camp Douglas.—The following letter was received too late for insertion in its proper place, under the head of Army Correspondence, and is of too much interest to be deferred:—

### SANITARY CONDITION OF CAMP DOUGLAS.

### CORRESPONDENCE OF PROF. J. H. HOLLISTER, ONE OF THE CONTRACT SURGEONS OF THE POST.

Editor of the Examiner. *Dear Sir:*—It may interest your readers to learn more fully the condition of the prisoners confined at this place, and the character of the diseases from which they suffer, than may be gleaned from occasional paragraphs in the secular papers.

Let me premise, by a word of reference to the place and to those in command, before I speak of the prisoners and their condition.

Camp Douglas, so named from its immediate proximity to the beautiful grove, three miles south of our city, formerly the property, and bearing the name, of the late Senator, was fitted up for rendezvous and instruction of our own soldiers, and occupied by several thousands of them, till called to the active duties of the field. It covers an area of about 40 acres, so situated as to have the full effect of the counter currents of lake and prairie winds, and though lower in position than might be desired for camping grounds, its sandy subsoil permitted the rapid disappearance of the surface water, upon the cessation of the heavy spring rains, and soon rendered it perfectly comfortable for occupation.

*The Command* of the post since the quartering of the prisoners, who arrived here immediately after the fall of Fort Donelson, has devolved upon Col. Mulligan's Irish Brigade, the 23d Ill. Vols. assisted by Col. Cameron, with his Scotch Regiment, the 65th.

In every respect, the oversight and control has been gentle but firm, and the two regiments, though severely taxed by such extended sentry duties, have nobly accomplished their labors.

*The Medical Attendants* of the sick in camp have been composed of the surgeons of the regiments referred to and their assistants; of surgeons employed by special contract; and such of the Confederate surgeons as were found competent to the duties assigned.

While the camp was under the jurisdiction of the State, E. ANDREWS, Professor of Surgery in Lind University, was post-surgeon. When the care was assumed by the U. S. Government, Surgeon WINER, of Col. Mulligan's Brigade, succeeded to the position, being the senior surgeon of the regiments, and has occupied this position during my connection with the camp. It is but simple justice to him to state that he has met the arduous duties of that position promptly, ably, and successfully, proving himself *another* of the right men in the right place. His assistant-surgeon, TAYLOR, a graduate of Lind University, by his unusual efficiency and urbanity of manner, in accomplishing the laborious details incident to his position, and by his untiring vigilence and care for the sick, has rendered himself emphatically *the pet of the camp.*

To say *more* of Col. Mulligan's Medical Staff would seem undue laudation, to say *less* would do them injustice.

Dr. PARKS, of our city, whose acquaintance I made in camp, is the surgeon of Col. Cameron's Regiment, and has had special supervision of the wards of our own sick soldiers. He is well posted, and competent to the emergencies to which a regimental surgeon, in active service, is momentarily liable.

*The Contract Surgeons,* so far as I know them, were the lamented ROCKWELL, who too soon fell a victim to over-work and his sympathy with the suffering, and JNO. M. WOODWORTH, another indefatigable worker—since appointed to a prominent position in the army below Corinth. These gentlemen both graduated with great credit to themselves and the school at the last commencement of Lind University.

Besides these, Dr. THOMAS BEVAN, of our city, and myself, were in daily attendance upon the wards assigned to our charge.

Of the *Confederate Surgeons* I have to state that I became much interested in those with whom I had professional relation.

To these men was committed the care of the invalids in the barracks previous to their entrance to the hospital wards—though, latterly, several of them have rendered efficient service in the hospitals, assuming the entire oversight, as prescribing physicians of several of the wards. Among those thus engaged are Drs. Kennedy, Foxie, Reid, Wood, Duprie, Williams, and several whose names, at this moment, I do not recall. They have been faithful to the wants of the sick, promptly and cordially co-operating with the post-surgeon in all matters of sanitary interest.

*The prisoners* have the appearance of all armies long in service without change of their heavier clothing. They are almost entirely without uniform; and among the divers colors of dress, one is so decidedly preponderant as to earn for the prisoners the sobriquet of "the butternuts,"—although the *walnuts* would be more appropriate.

As fighting material, in physical and mental development there are many among them equal to those of any age or nation. Still the very large preponderance of *delicate*, *pale*, and *frail* ones—many of them mere boys in years and growth—places them in strong contrast with the regiments of our own and adjoining States, of many of whom I am able to speak from personal observation.

There have been additions to the rebels first quartered here, by arrivals from Island No. 10, from Pittsburg Landing, from the convalescent wards of the St. Louis hospitals, and finally, by the return of those originally conveyed to Madison, Wisconsin; so that now the whole number in camp, I think, is not far from 10,000. The abuses which crept into camp in consequence of the very free access of citizens, and the friends of those in confinement, rendered it necessary to exclude all but those whose duties were imperative to the proper care of the place. The duty of the command has been strictly and impartially discharged, and those evils corrected.

*The hospitals* are similar in construction to the barracks, built of upright boards, closely battoned, the roof being rendered very perfect by the use of cement composition.

The buildings are subdivided, each, into from two to four wards or rooms, about 20 feet wide, and from 40 to 80 feet long. The sick are ranged on either side of the rooms, leaving broad central aisles through their whole length. The camp cot, with mattress, blankets, sheet, and pillow, complete the bed furniture, while an ample supply of drinking cups, spittoons, &c., renders each person independent of the others, as regards these necessities of the sick-room. The cots, and the space which each should occupy, are numbered. The number is placed prominently on the wall, and beneath it a hook, upon which hangs either a slate or sheet of paper. Beneath this a shelf is placed for the drinking cup, spoon, and medicines for the patient answering to that number, and occupying that cot. The space allotted to each patient is about six feet, and from 16 to 35 are appointed to a single ward. Upon the slate the name of the patient is registered; his company and regiment are also recorded, and beneath these, the daily prescription of the attending physician is written; and thus not only does he remember the history of the case at a glance, but in his absence, any assistant could follow him, knowing just what had been previously prescribed. The directions for the nurse are written immediately beneath the prescription, and the date carefully noted in the margin. When the physician has completed his round, the slates or papers (each numbered) are taken to the dispensary, where the prescription of each is filled and numbered. The slates and medicines are then returned, and all placed according to their respective numbers, and the nurse finds above each bed the medicine for that patient, and the written directions for its use. I have been purposely minute that this system of prescribing might benefit those not familiar with hospitals, and who in such times as have now fallen upon us, may be called to the discharge of hospital duties, without previous experience. The wards are 13 in number, and contain 330 cots. About 40 to 60 patients are assigned to a single physician for treatment. It is usual for an older physician to have oversight of three to four wards, with the aid of an assistant physician with whom to divide the labors. To each four wards, there are detailed a ward-master, to whose care the clothing of patients is committed during their sickness, and who is responsible for the performance of all requirements of the physician; and for each ward six nurses: four for daily, and two for night service of the sick. The duties of these pertain to the proper temperature of the rooms, the assistance and supply of the sick, covering of those sleeping, and the immediate removal of all offensive matters from the room.

The physician orders the diet of invalids, and the steward is governed by his requisition in supplying them. There is a general *ration-list* for the sick room, to which the cooks conform, unless by special orders, particular diet is required. It will thus be seen that for the comfort of our sick prisoners every thing has been done that an enlightened humanity could reasonably dictate or demand; and I desire to make this public record of facts, that there may be no misjudgement of their treatment, by those who wonder at the mortality in camp, or are prejudiced against our Northern people.

I come now to speak of the *diseases* of the camp. No medical man will pass through the wards without expressing surprise at the great similarity of their diseases. But when we reflect upon the similarity of their former methods of life, climate, &c. their uniform *food, dress, and exposures* in camp-life, we are not so much surprised that like causes should so invariably produce similar results.

The patients are presented to us for treatment after from 4 to 12 months' service,—some after enduring almost incredible hardships, as in the case of those lying in the trenches at Fort Donelson, many of them two days without food, and constantly exposed to rain and snow. Still worse, as at Island No. 10, where men by thousands were in the open field, without tents, for three weeks, and with no protection but their blankets. With such precedent exposures it is not singular that they should present themselves in anything but a desirable condition for treatment, or that when finally they become prostrated there should appear so little recuperative power to aid them in recovery!

The twofold influences of a warmer climate and such severe exposures have had the effect to render them extremely *anœmic*, and this condition gives *character* to all their diseases.

Nine-tenths of the inmates are affected either by pneumonia or chronic diarrhœa, and in about one-third of the cases there is a complication of the two diseases.

*Pneumonia*, as here presented, has symptoms very similar to those in our own latitude, where the patient, during the previous year, has suffered severely from malarious disease, with marked impoverishment of the blood, and, encountering a severe winter, is prostrated in February or March with an asthenic form of this disease.

In many instances, the patients have been unwell for one, two, and even three months, with a "powerful" cough, or "right smart" raising. The day before their admission to the hospital they had severe chills, and are now complaining bitterly, in most instances, of pain as of *pleuro-pneumonia*, now frequently in the left side; and in about one-fourth of the cases presenting well-marked double pneumonia. For the time being, the pain, upon motion of the chest, is the predominant and most aggravated symptom. It yields very tardily to ordinary counter-irritation or dry cupping. In 50 cases, at least, I have witnessed relief in from 10 to 15 minutes, from the application of from 3 to 5 wet cups, over the point of pain, and in almost every instance the relief has been permanent. In these cases we have not facilitated the flow of blood, and the patient seldom loses more than from two to four ounces. We are convinced that this moderate depletion is more than atoned for by the immediate results. The sthenic symptoms usually subside in from 24 to 36 hours after the onset of the disease; and the use of stimulating and sustaining treatment become immediately imperative.

In almost all instances, there is passive congestion of one or both lungs, and while there is tolerable resonance upon percussion of the anterior portion of the chest, while the patient is recumbent, if, changing his position to a sitting posture, the posterior walls are examined there is in almost all cases a very marked dulness, and usually entire absence of respiratory murmur in the posterior portion of one and very frequently of both lungs. The tongue is almost never *dry* nor *coated;* a slight redness being frequently present, and the salivary secretion rather excessive than otherwise. Not one patient in twenty suffers from constipation; not one in ten has the icterie appearance of the skin. The pulse is usually feeble and frequent, and very frequently has the same characteristics as in the second stage of typhoid fever. The progress of the disease is indicated by increased dulness, showing an increase of pulmonary congestion by greater difficulty of respiration, palor of the body, slight lividity of the extremities, and that uniform prostration which indicates a fatal termination not far distant. The progress of the disease is usually very rapid, and the termination sometimes so sudden as to impress us with the utter want in their systems of any recuperative energy to withstand the shock.

The sputa is usually copious, not very consistent, and not in one case in fifty has it the rusty appearance. In later stages, and in the severer cases of longer standing, it is markedly muco-purulent. *The treatment*, as before stated, from the first, has reference to sustaining the vital powers and equalizing the circulation.

For this purpose, while in the first stage, the pulse being strong and frequent, veratrum is sometimes given for its temporary control, still quinine, combined with a little opium, is not unfrequently the very first prescription; nor can it be withheld till the patient is fully convalescent. Complicated as this disease is, with diarrhœa, a favorite pill for its treatment is composed as follows:—℞. Sulph. quinia,
Piper nigri, pulv.
Tannin, aa. gr. i.
Pulv. opii, " ss. M. Ft. Pil.

One to be given every four hours till discharges are checked, when the pepper and tannin are omitted, and the opium diminished, while the quinine is increased. Friction over the bowels with ol. terebinth, seems frequently to benefit the patient, as also the exhibition of the turpentine emulsion, in small doses, internally.

Blisters behave so badly as to be contra-indicated. If once the cuticle is removed, there is usually such copious seropurulent discharge, and such difficulty in healing them, that in most cases I am led to abandon them, using in place stimulating embrocations and hot fomentations. In several cases I was induced to try the effect of alcoholic stimulation alone, and under the influence of milk punch, given every one or two hours, I frequently saw the pulse diminish, the congestion subside, and the patient convalesce. The after-treatment, of course, was more strictly tonic. My views of the use of alcohol I have not space in this already too long letter to give; but I firmly believe that I saved a nu nber of patients which I should have lost, but for *stimulation*, followed by tonics and iron.

My attention was early called by Dr. WINER, to the use of Donovon's solution (liquor arsenici et hydrarg yri iodide,) in the treatment of pneumonia. I watched its effects in about 75 cases, as many as 40 I treated with this medicine alone, in connection with a nutritious diet, and I must say that in most instances, I was charmed by the result. The tonic effect of the arsenious element and its stimulation of the capillary system fulfilled an important indication. The mercurial alterative was here exhibited sufficiently to fully meet the necessity of the case, while the glandular stimulation of the iodine seemed to complete the requirement.

It is contra-indicated where there is decided irritability of the stomach and bowels, or where in the advanced stage of the disease the system is greatly prostrated. The dose was from 10 to 30 drops in milk every two hours.

The diarrhœa indicated sometimes the mere atony of the mucous membrane of the bowels; more frequently, as the *post mortem* examinations showed, there was a chronic enteritis, which yielded but little to astringents, and by which in fact it was more frequently aggravated. Bland nutritious food, and the sub-nitrate of bismuth combined with opium, seemed best to control the difficulty. Dropsical effusions are a very frequent concomitant or sequal of these diseases. Erysipelatous inflammation of the face and extremities very often supervene, but yielded as readily to muriate tinct. of iron, as ague does to quinine.

Pardon the length of my letter, and give but an extract if your limits require. Respectfully yours.

J. H. HOLLISTER.

*Camp Douglas, June* 1, 1862.

---

CHANGES IN THE MED. DEPART. OF LIND UNIVERSITY.—On account of continued ill-health, Prof. A. L. MCARTHUR has resigned the chair of Materia Medica and Therapeutics in this Institution. Prof. MCARTHUR was able to give but little more than half of his course last winter, the deficiency being supplied by other members of the Faculty.

Prof. MCARTHUR was an earnest and interesting lecturer, and a highly esteemed member of the Faculty. It is greatly to be regretted, that the promising career of usefulness open before him, should be so soon checked by protracted physical infirmities.

The chair of Materia Medica and Therapeutics, made vacant by the resignation of Prof. MCARTHUR, has been filled by Prof. J. H. HOLLISTER, transferred from the chair of Descriptive Anatomy. The latter chair has been filled, temporarily, by the appointment of J. S. JEWELL, M.D., Lecturer on Descriptive Anatomy and Demonstrator. Dr. JEWELL, was a member of the first graduating class of the University, and will discharge the duties of his new position with a zeal and ability, that will soon earn for him an enviable reputation.

---

CHANGES AT CAMP DOUGLAS.—Dr. WINER, surgeon to the 23d Regiment of Illinois Volunteers, (Irish Brigade), having left with his regiment for the seat of War in Virginia, the office of Post-Surgeon has been assigned to Dr. B. MCVICKAR, of this city, aided by a number of assistants.

The number of prisoners in the Camp, at this time, is about 9000, of whom little more than 300 are on the sick list. The principal diseases are typhoid fever, diarrhœa, pneumonia, and erysipelas.

Army Medical Officers not Belligerents.—We are very glad to learn that our Government has adopted the principle, that Surgeons and Assistant-Surgeons, are not belligerents in war, and consequently not to be taken or held as *prisoners.* The common interests of humanity require that the Surgeon should be allowed to administer freely to the sick and wounded, wherever found on the field of strife, without liability of arrest or imprisonment.

---

### NEW YORK ACADEMY OF MEDICINE.

### DISCUSSION OF DR. J. M. SIM'S PAPER ON VAGINISMUS.

Dr. Peaslee.—Mr. President, I feel for one under very great obligations to Dr. Sims for giving to this disease a distinctive name, and I think the whole profession, so far as they are acquainted with the disease, will feel a similar obligation. In regard to the use of precise terms employed by Dr. Sims, I think the termination indicates the nature of the disease. In regard to the recommendation of the last speaker (Dr. Stevens), I think that many a woman who had suffered as in the first case related, would rather die than have a continuance of the pain. The first case that came under my notice was recognized by mere chance, and occurred some ten years since, in a lady who had been married eleven months. In that instance the husband was not wanting in efforts on his part, neither was the wife wanting in patience and endurance on hers—the sexual act, however, was never accomplished. I was applied to for advice, and found the lady in the condition of a "nervous wreck," as Dr. Sims styled it. On examination, I found what I supposed to be a partial occlusion of the vagina by the hymen, and I accordingly proposed an operation for a divison of the membrane. It was about the time when ether was commencing to be used for anæsthetic purposes, and the sensitiveness of the parts was so great, that I remarked that I could not perform such an operation without first inducing insensibility. I gave the ether, and to my astonishment found that it was very easy to introduce the finger into the vagina, the former resistance to such an endeavor being now removed. I hence referred the difficulty to spasm of the vagina which was confined to the sphincter muscle. I made use of unguents, among which was one composed principally of extract of belladonna, which seemed to relieve the sufferings to that degree that the sexual act was accomplished after a time. The patient resided in the State of Maine, was under treatment but a short time, and I have not heard anything from her since. Within about five years after, I saw another case precisely similar in character, though with less severe symptoms. I may here remark, that I believe cases may be met in which there is every gradation, from the severity of the symptoms in Dr. Sims's first case, down to those in which but slight hinderances to sexual intercourse exist. The case I now refer to was a lady who never had children, who had been married a period of ten years, who had frequently suffered from sexual intercourse, but who, some months previous, had found the accomplishment of the act impossible, without the greatest agony. In that instance, by using an ointment, composed of two grains of atropine to ℥j. of lard, I succeeded in overcoming the spasm in about a fortnight. She remained well for two years, when I was again applied to, and the same treatment was available. I have seen quite a number of cases of Vaginismus, and I have been able to relieve all thus far, with the exception of my first case, which I had in charge only a short time. The ointment which I have generally used has been composed of atropine and lard in the proportions mentioned. I, of course, did not limit myself to the exclusive use of this remedy, but also employed mucilaginous injections, or injections containing extract of hyosciamus. As soon as the disposition to contraction is overcome to that extent as to make it allowable, I make use of a small-sized dilator. I have seen a case of vaginismus within the last fortnight—a lady had been married seven years, but had enjoyed sexual intercourse only about twice. It is possible now to introduce the index finger into the vagina, and in this instance, as in all the cases which I have seen, there is the excessive tenderness of the hymen, or carunculæ myrtiformes, as described by the author of the paper. In conclusion, Dr. P. asked concerning the extent of the incisions made by Dr. Sims.

Dr. Sims.—The incisions I make are more in the form of a Y than anything else. I commence first on the right of the middle line, about half an inch above the margin of the sphincter muscle. The sphincter muscle is about half an inch across, and from its edge down to the outlet of the perineal opening, where the skin becomes mucous membrane, is very nearly an inch in most women. My incisions meet just below the lower edge of the sphincter muscle, and become one incision down to the outer edge of the skin. In regard to the composition of the term vaginismus, I think there are very many comprehensive words used in medicine which are made up of mixture of Latin and Greek. However, I care very little by what precise name the disease is called; only it strikes me that the term is a comprehensive one, and that every physician who is not a good classic scholar will not be under the necessity of looking up the meaning of it in his Lexicon. In regard to the application of belladonna—one of my patients had used the ointment for years without any good effect, and the case now under my care, upon which I have not yet operated, has also proved the inefficiency of the remedy. The operation which I propose cures the disease, but the use of the dilator makes the cure permanent.—*American Medical Times.*

---

TAKING COLD, ETC.—In Dr. Ware's tenth lecture upon General Therapeutics, concluded in the *Boston Medical and Surgical Journal* for January 30, remarks are made upon the abstraction of heat from the body, "taking cold," etc. Dr. Ware refers to the solicitude of many in avoiding exposure by which colds are taken, and remarks very justly, we think, that

"The greatest error, perhaps, on this point, arises from the disposition to substitute external warmth by artificial heat, and the exclusion of air and confinement within doors, for adequate clothing. The effect of this error is to deprive patients with chronic diseases, and even persons with simply feeble health, of the invigorating influence of external air. Bad and variable as our climate is, there are few individuals who may not, by the careful and gradual cultivation of the habit, acquire the power of going abroad at all seasons, and almost all weathers, not only without danger, but with positive benefit. More persons induce imperfect health by the habits of seclusion engendered by the *fear* of taking cold, than are actually injured *by* taking cold. No doubt an artificial state of the system in this particular is produced in many persons, and no sudden change of habit would be safe."

Our experience justifies, and we have long entertained opinions as above. Those persons who are the most particular to observe all cautions against taking cold, by keeping indoors, except on warm and sunny days, are by far the more likely to suffer from colds than those who have more energy and boldness in exposing themselves. The rose-bush reared in the open air will flourish and bloom in its perfection of beauty. A similar one reared in a blinded and curtained sitting-room or parlor, if carried into the open air, will wilt when the sun looks brightly upon it, or shiver and die at the touch of the first white frost. Almost everybody supposes that winters are particularly unfavorable for consumptive invalids. This should not be so. The tonic influence of cold, its power to increase the appetite, and the function of assimilation, should increase the strength, and add vigor to the nervous system. Besides, the greater density of the atmosphere should enable the lungs, in their diseased condition, causing diminished availability, to do their required labor and perform their requisite functions easier and better than in summer, when the atmosphere, per cubic foot, from its rarity, contains far less oxygen. If the consumptive patient has too little energy to expose himself to the winter's cold, or is too timid to let the lungs feel the influences of the dense and pure air, and shuts himself up in the tight, artificially warmed room, where even the rarified atmosphere is impure, then are winters peculiarly trying to consumptive patients.—*Medical & Surgical Reporter.*

---

The Act re-organizing the Medical Department of the Army provides for the appointment of eight Sanitary Inspectors, whose special duty would be to visit the camps and hospitals, and supervise their sanitary condition. These appointments have been delayed, to the great detriment of the army. The nominations have finally been made, and the Senate has acted upon them. So far as announced, the following gentlemen have been selected, viz.: Dr. John M. Cuyler, Surgeon, U.S.A.; Dr. Richard H. Coolidge, Surgeon, U.S.A.; Dr. Edward P. Voldeum, Assistant Surgeon, U.S.A.; Dr. Geo. H. Lyman, Geo. F. Allan, and W. H. Mussey, Brigade-Surgeons, U.S.A.—*American Med. Times.*

---

Surgeons and Nurses.—The demand is and will continue to be for competent surgeons and nurses. They are wanted not temporarily but permanently. New surgeons offering their services should do it with this reference. If volunteers cannot be obtained, Dr. Cuyler is prepared to hire competent surgeons, who will be expected to engage themselves as long as their services are required.

By a recent Act of Congress, the rank of Brigade-Surgeon is abolished, and this class of medical officers are subject to the same rules which govern surgeons. The corps of Surgeons is also to be enlarged by the appointment of 160 more for the war, 40 being full Surgeons, and the remainder Assistant-Surgeons.

At the Annual Meeting of the Medical Society of the State of Pennsylvania, held in Philadelphia, a committee was appointed to inquire as to the expediency of publishing a Daily Medical Gazette.—*American Medical Times.*

